# The fasting blood glucose and long non-coding RNA SNHG8 predict poor prognosis in patients with gastric carcinoma after radical gastrectomy

**DOI:** 10.18632/aging.101576

**Published:** 2018-10-06

**Authors:** Yunchai Lin, Dan Hu, Qiang Zhou, Xiangdong Lin, Jinxiu Lin, Feng Peng

**Affiliations:** 1 Department of Cardiology, The First Affiliated Hospital of Fujian Medical University, Fuzhou, Fujian, China; 2 Department of Pathology, Fujian Cancer Hospital and Fujian Medical University Cancer Hospital, Fuzhou, Fujian, China; 3 Department of Endocrinology, The Second Hospital of Fuzhou, The Affiliated Hospital of Xiamen University, Fuzhou, Fujian, China; * Equal contribution

**Keywords:** fasting blood glucose, gastric carcinoma, SNHG8, prognosis, mortality

## Abstract

This prospective study sought to evaluate the prediction of fasting blood glucose and long non-coding RNA (lncRNA) SNHG8 for the risk of gastric carcinoma mortality. A total of 217 gastric carcinoma patients underwent radical gastrectomy were included during 2012-16. The final follow-up was finished in January 2017. The aggregate hazard ratio(HR) demonstrated that poor prognosis of gastric carcinoma was associated with fasting blood glucose (HR= 1.29, P=0.037), SNHG8 expression(HR = 1.10, P= 0.009), positive distant metastasis(HR = 2.99, P= 0.020), EBV positive (HR = 3.40, P=0.002), and tumor size more than 5.0 cm (HR = 3.36, P= 0.005). In survival analysis, elevated fasting blood glucose (P =0.007) and high SNHG8 expression (P =0.007) were significantly associated with shorter survival times in gastric cancer. Significant multiplicative interaction was shown between fasting blood glucose and SNHG8 expression (chi-squared=7.81, P_multiplicative_ =0.005), without statistical additive interaction. Fasting blood glucose and SNHG8 expression could predict poor prognosis after radical gastrectomy. LncRNA SNHG8 could be applied as a novel epigenetic molecular target in gastric carcinoma.

## Introduction

Gastric carcinoma with high mortality had become a major public health problem worldwide [1]. In China, gastric carcinoma was the most common malignant tumor and the second leading cause of malignant tumor death [2]. Epstein -Barr virus-related gastric carcinoma was a unique subtype accounting for about 10% [3]. Due to the lack of specificity of symptoms, most of patients with gastric carcinoma were found in the middle or late stages, and their prognosis was often poor [4]. The clinical outcomes of patients with similar clinical stage and treatment were often different [5]. Therefore, in order to improve the survival rate, it was clinically necessary to identify prognostic risk factors and effective biomarkers to provide a more accurate prognostic assessment.

The potential predictive risk of diabetes in malignant tumors had been extensively studied. A meta-analysis based on 97 prospective studies revealed a doubling of the risk of mortality in cancer complicated with diabetes [6,7]. Lindkvist et al. [8] pointed out hyperglycemia was considered to be a promising risk factor for gastric carcinoma in women. In addition, meta-analysis including 4 case-control and 17 cohort studies [9] showed diabetes significantly increased the mortality of gastric carcinoma in a follow-up period of more than 10 years. Another large cohort study indicated diabetes was considered as a vital factor in the development of gastric carcinoma [10]. Moreover, previous FIESTA [11] had demonstrated hyperglycemia could predict a worse prognosis after radical gastrectomy, especially in the early stage. Appropriately screening methods should be a pivotal part of the clinical management of diabetes patients. Thus, strengthening early screening of blood glucose would effectively improve the prognosis of gastric carcinoma and prolong the survival of patients.

Long noncoding RNA (lncRNA), a kind of RNA molecules with a transcript more than 200 nucleotides but without a complete open reading frame, once was regarded as a noise of genomic transcription [12]. In recent years, lncRNA had been found to act as a gene expression regulator and affect the progression of cancer [13,14]. SNHG8 at 4q26 encoded a novel small nucleolar RNA and participated in a number of biological functions such as translation, transcription, regulation of transcription and RNA splicing [15,16]. The lncRNA SNHG8 had been reported to be dysregulated in gastric cancer and promote tumor invasion or metastasis as a proto-oncogene [17]. In conclusion, SNHG8 could be applied as a biomarker for poor prognosis of gastric carcinoma.

Based on the previous sequence of lncRNA SNHG8, we evaluated the association between fasting blood glucose and SNHG8 expression on the prognosis of gastric carcinoma patients, as well as the interaction. It aimed to seek a new biomarker for predicting clinical outcomes of gastric carcinoma patients.

## RESULTS

### Baseline characteristics

The clinical baseline features and SNHG8 expression in gastric carcinoma patients were compared in Table 1. Data were described as median (interquartile range) or percentage. There was no significant difference in age, gender and smoking between non survival group and survival group (P > 0.05). The two groups were similar in clinical characteristics such as systolic blood pressure (SBP)(P = 0.160), diastolic blood pressure(DBP) (P = 0.453), total cholesterol (TC)(P = 0.730), high-density lipoprotein cholesterol (HDL) (P = 0.113), and low-density lipoprotein cholesterol (LDL)(P = 0.642). Fasting blood glucose was significantly elevated in the non -survivors (P = 0.012). In contrast, body mass index (BMI) and triglycerides of the non-survivors were significantly lower (P = 0.001). Notably, the expression of SNHG8 and EBV positive in the non-survivors were higher than those in the survivors (P = 0.002 and P < 0.001, respectively).

**Table 1:** The clinical baseline characteristics of non-survivor and survivor patients.

Characteristics	Total N=217	Non-survivors N=46	Survivors N=171	P value
Age(years)	59(51,65)	58(45,67)	59(54,65)	0.374
Males	163(75.1%)	31(67.4%)	132(77.2%)	0.122
Smoking	48 (22.1%)	12 (26.1%)	36(21.2%)	0.293
BMI(kg/m^2^)	23.03(20.98, 25.21)	21.56(20.93, 24.61)	23.12(21.48, 25.31)	0.041
SBP(mmHg)	120(114,134)	123(116,145)	120(114,132)	0.160
DBP(mmHg)	78(70,84)	80(70,85)	78(70,83)	0.453
FBG(mmol/L)	5.11(4.66,5.81)	5.64(4.97,6.60)	5.07(4.62,5.71)	0.012
TG(mmol/L)	1.06(0.73,1.45)	0.78(0.65,1.40)	1.16(0.77,1.45)	0.001
TC(mmol/L)	4.69(3.99,5.17)	4.78(3.71,5.15)	4.69(4.13,5.23)	0.730
HDL(mmol/L)	1.24(1.08,1.48)	1.24(0.95,1.40)	1.25(1.09,1.59)	0.113
LDL(mmol/L)	3.20(2.47,3.56)	3.27(2.47,3.73)	3.19(2.48,3.56)	0.642
SNHG8 expression	2.15(1.17,3.74)	3.07(1.26,8.59)	1.89(1.16,3.10)	0.002
EBV(+)	87(40.1%)	30(65.2%)	57(33.3%)	<0.001
TNM stage				<0.001
I-II	57(26.3%)	3(6.5%)	54(31.6%)	
III-IV	160(73.7%)	43(93.5%)	117(68.4%)	
Differentiation				0.002
Moderate/High	73(33.6%)	7(15.2%)	66(38.6%)	
low	144(66.4%)	39(84.8%)	105(61.4%)	
Distant metastasis	118(54.4%)	37(80.4%)	81(47.4%)	<0.001
LNM	154(71.0%)	43(93.5%)	111(64.9%)	<0.001
Lauren’s classification				0.002
Intestinal type	73(33.6%)	7(15.2%)	66(38.6%)	
Diffuse type	144(66.4%)	39(84.8%)	105(61.4%)	
Tumor size (cm)				<0.001
Maximum diameter >5	108(49.8%)	36(79.3%)	72(42.1%)	

Abbreviations: BMI=body mass index; SBP=systolic blood pressure; DBP=diastolic blood pressure; FBG= fasting blood glucose; TG= Triglyceride; TC=total cholesterol; HDL= high-density lipoprotein cholesterol; LDL=low-density lipoprotein cholesterol; EBV= Epstein-Barr virus; TNM=tumor node metastasis; LNM= lymph node metastasis. P was calculated by Mann-Whitney U Test or Chi-square test where appropriate.

In addition, remarkable difference was detected between two groups in tumor TNM stage (p < 0.001), differentiation (p =0.002), distant metastasis (p < 0.001), lymph node metastasis (p < 0.001), and Lauren’s classification (p = 0.002). The size of tumor (maximum diameter >5cm) was significantly larger in non-survivors (p < 0.001).

### Association of clinical factors and gastric carcinoma mortality

We explored the relationship between clinical factors and mortality of gastric carcinoma, including fasting blood glucose, BMI, SBP, DBP, TG, TC, HDL, and LDLC (Table 2). After adjusting for age, gender, smoking, tumor size, TNM stage, Lauren’s classification and differentiation. Among those eight clinical factors, the increase risk of gastric carcinoma mortality was moderately related with elevated fasting blood glucose (HR = 1.32, 95% CI: 1.01–1.72, P = 0.040) (Table 2). The mortality rate increased by nearly 32% with an adding of per one standard deviation in fasting blood glucose. Despite P value less than 0.05, TG(HR = 0.47, 95% CI: 0.26-0.87, P = 0.015) (Table 2) was negatively correlated with the risk of gastric carcinoma death.

**Table 2:** Univariate analysis of clinical factors and risk of gastric carcinoma mortality.

	Increment (s.d.)		HR, 95% CI, P		HR, 95% CI, P*
BMI	-0.05kg/m^2^		0.95, 0.87-1.04, 0.293		0.98, 0.88-1.09, 0.700
SBP	0.11mmHg		1.01, 0.99-1.03, 0.481		1.01, 0.99-1.03, 0.367
DBP	0.11mmHg		1.01, 0.98-1.04, 0.467		1.03, 1.00-1.07, 0.087
FBG	0.27mmol/L		1.31, 1.04-1.66, 0.024		1.32, 1.01-1.72, 0.040
TG	-0.61mmol/L		0.37, 0.18-0.78, 0.009		0.47, 0.26-0.87, 0.015
TC	-0.05mmol/L		0.96, 0.74-1.23, 0.723		1.32, 1.00-1.74, 0.053
HDL	-0.36mmol/L		0.34, 0.14-0.80, 0.014		0.90, 0.33-2.48, 0.903
LDL	0.04 mmol/L		1.04, 0.75-1.45, 0.794		1.36, 0.99-1.87, 0.058

Abbreviations: s.d.=standard deviation; HR=hazard ratio; 95% CI=95% confidence interval; BMI=body mass index; SBP=systolic blood pressure; DBP=diastolic blood pressure; FBG= fasting blood glucose ;TC=total cholesterol; HDL= high-density lipoprotein cholesterol; LDL=low-density lipoprotein cholesterol. The effect estimates were evaluated through Cox regression model. P*: age, gender, smoking, tumor size, TNM stage , Lauren’s classification and differentiation were adjusted.

### Association of clinical pathologic characteristic and gastric carcinoma mortality

Increased risk of gastric carcinoma mortality was closely associated with high expression of SNHG8 (HR= 1.10, 95% CI: 1.03–1.19; P=0.009), distant metastasis (HR= 2.99, 95% CI: 1.19–7.50; P=0.020), tumor size more than 5.0 cm (HR= 3.36, 95% CI: 1.48–8.96; P=0.005), and EBV-positive (HR= 3.40, 95% CI: 1.57–7.37; P=0 .002). However, there was no significance in Lauren’s classification, differentiation, TNM stage, and lymph node metastasis. The result revealed high SNHG8 expression was significantly associated with poor prognosis in gastric carcinoma patients.

### Survival analysis of SNHG8 expression for gastric carcinoma mortality

**Figure 1:**
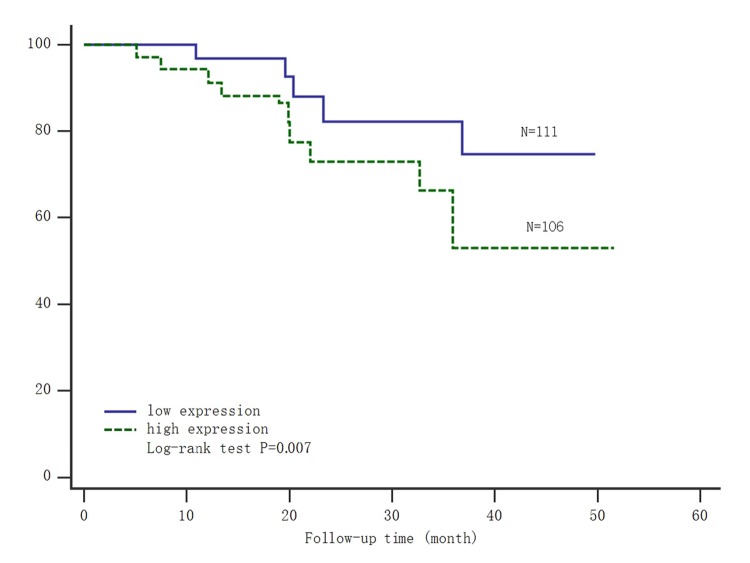
**Kaplan-Meier survival analysis of the expression of SNHG8 and gastric carcinoma mortality.** Abbreviations: low expression=low expression level of SNHG8; high expression=high expression level of SNHG8.

The patients were divided into high and low SNHG8 expression group, according to the expression level of SNHG8 through quantitative polymerase chain reaction (PCR). The Kaplan–Meier survival analysis and log-rank test were used to evaluate the relationship between SNHG8 expression and prognosis of gastric carcinoma patients. From the survival curve, we observed patients with high level of SNHG8 expression had significantly shorter survival time (log-rank test P =0.007; Figure 1). It suggested lncRNA SNHG8 could be recognized as an independent prognostic factor.

**Figure 2:**
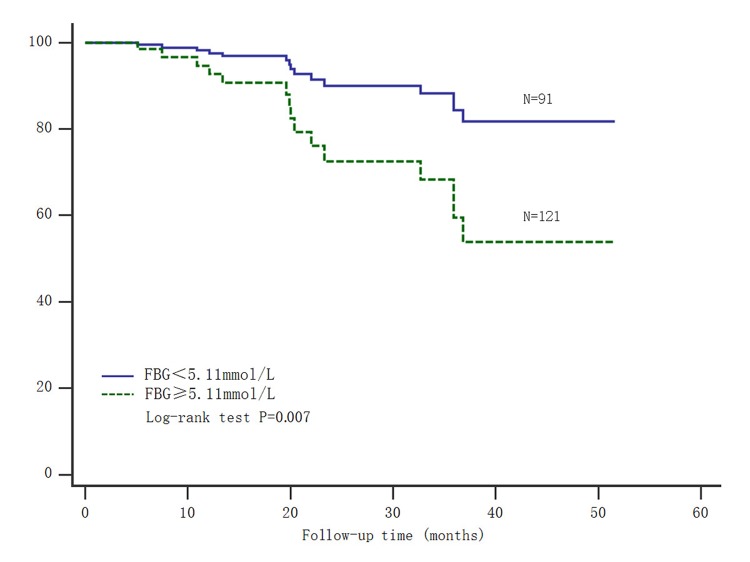
**Kaplan-Meier survival analysis of fasting blood glucose and gastric carcinoma mortality.** Abbreviations: FBG= fasting blood glucose.

Moreover, gastric carcinoma patients were also divided into two groups based on the median of fasting blood glucose. Patients with fasting blood glucose ≤ 5.11 mmol/L had significantly longer survival time (Log-rank test: P=0.007) (Figure 2). Thus, both of high SNHG8 expression and elevated fasting blood glucose could predict poor prognosis for patients after radical gastrectomy.

### The interaction of fasting blood glucose and SNHG8 expression

**Figure 3:**
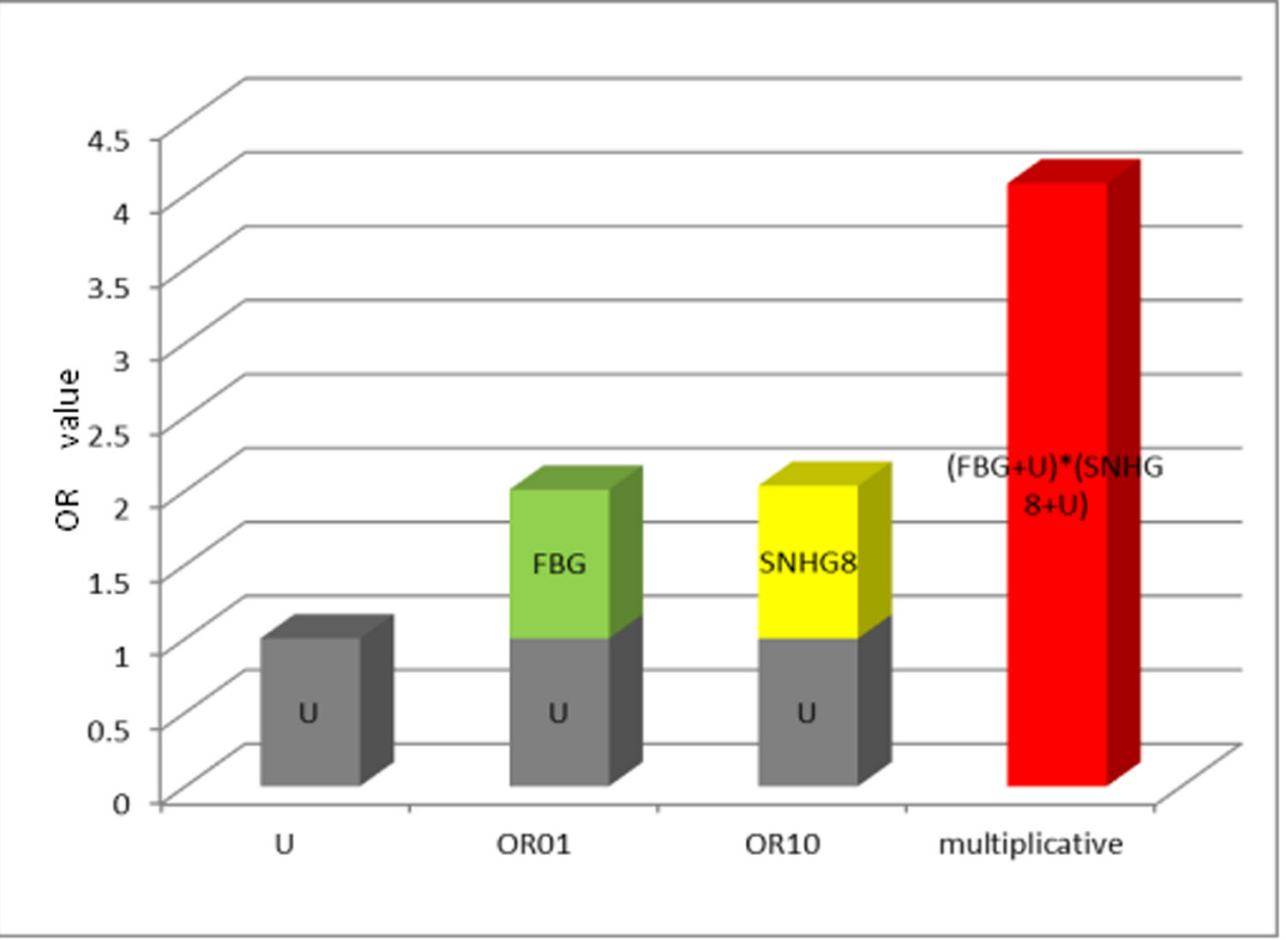
**The multiplicative model histogram of SNHG8 expression and FBG.** Abbreviations: OR= odds ratio; FBG= fasting blood glucose; OR01 referred to only exposure to fasting blood glucose, OR10 referred to only exposure to SNHG8, and U=1 was set as a control group indicating no exposure.

After adjustment for age,gender and smoking by multivariate cox regression model, significant multiplicative interaction was shown between SNHG8 expression and fasting blood glucose on gastric cancer mortality (chi-squared[X^2^] =7.81, P_multiplicative_=0.005) (Table 4). From Figure 3 , the OR value of the interaction indicated lncRNA SNHG8 and fasting blood glucose had a multiplicative interaction in predicting poor prognosis of gastric cancer.

**Table 4:** The multiplicative interaction of fasting blood glucose and SNHG8 expression on Gastric Cancer Mortality.

	β	OR	95%CI	P
SNHG8	0.01	1.01	0.37-2.78	0.035
FBG	0.03	1.03	0.04-0.71	0.016
SNHG8 * FBG	0.52	1.69	1.17-2.44	0.005

Abbreviations: OR= odds ratio; 95% CI=95% confidence interval; FBG= fasting blood glucose

High expression level of SNHG8 and elevated fasting blood glucose could respectively increase the risk of gastric cancer mortality (P = 0.035 and P=0.016) (Table 5). When fasting blood glucose and SNHG8 expression were up-regulated simultaneously, the risk of gastric cancer mortality increased significantly (P = 0.001) (Table 5).

**Table 5:** The effect of fasting blood glucose and SNHG8 expression in Gastric Cancer Mortality.

SNHG8	FBG	β	OR	95%CI	P
0	0	-	1	-	-
1	0	0.01	1.01	0.37-2.78	0.035
0	1	0.03	1.03	0.04-0.71	0.016
1	1	0.47	1.89	0.28-1.28	0.001

Abbreviations: OR= odds ratio; 95% CI=95% confidence interval; FBG= fasting blood glucose;OR01 referred to only exposure to fasting blood glucose, OR10 referred to only exposure to SNHG8, OR11 referred to simultaneous exposure to fasting blood glucose and SNHG8, and OR00 was set as a control group indicating no exposure.

From table 6, the additive indexes including relative excess risk due to interaction (RERI), the attributable proportion due to interaction (AP) and the synergy index (S) with 95%CI between SNHG8 expression and fasting blood glucose were 0.65(-2.03, 3.32),0.39 (-0.63, 1.40) and 1.87 (0.01, 68.05) respectively. There was no statistical additive interaction, for the confidence interval of RERI and AP included 0, and S included 1.

**Table 6:** The additive interaction index of fasting blood glucose and SNHG8 expression.

	RERI			AP			S	
		point estimate	95%CI	point estimate	95%CI	point estimate		95%CI
SNHG8& FBG		0.65	-2.03, 3.33	0.39	-0.63, 1.40	1.87		0.01, 68.05

Abbreviations: OR= odds ratio; 95% CI=95% confidence interval; FBG= fasting blood glucose; RERE= relative excess risk due to interaction; AP=the attributable proportion due to interaction; S=the synergy index

## DISCUSSION

On the basis of 217 gastric cancer patients from this Prospective study, we considered the multiplicative interaction of fasting blood glucose and lncRNA SNHG8 on the survival of patients after radical gastrectomy. We found that multiplicative interaction was predictive of poor clinical outcome in gastric cancer patients with a median follow-up of 20.4 months. To our knowledge, this is by far the first study to evaluate poor prognosis of gastric cancer was associated with the multiplicative interaction of fasting blood glucose and lncRNA SNHG8.

Hyperglycemia was closely correlated with the carcinogenesis and development of gastric carcinoma. Their pathogenesis was a complicated process, possibly involving insulin resistance, chronic inflammatory response and abnormal expression of related cytokines, but the etiopathogenisis was unclear at present. Insulin, insulin-like growth factor 1(IGF1) and insulin-like growth factor 2(IGF2) were the most studied insulin-like peptides (ILPs) regarded as key regulators of energy metabolism and growth, especially IGF1 receptor and IGF2 receptor were expressed in gastric carcinoma cells [18–20]. Voluminous empirical evidences suggested insulin and IGF receptors mediated their effects on glucose transport and energy metabolism through signaling pathways downstream. Furthermore these receptors by binding insulin receptor substrate molecules promoted tumor cells proliferation, differentiation and metastasis, and simultaneously inhibited apoptosis [21–24]. Therefore, it was likely to antagonize insulin resistance by inhibiting these receptors, which was considered as a useful prophylactic and therapeutic strategy for cancer treatment [25]. In addition, Long-term hyperglycemia could provide energy source for malignant tumor cells as a nutrient, particularly for highly proliferating cancer cells. A large number of oxygen free radicals and acids were produced by glucose metabolism and had a direct tumor promoting effect [26]. We discovered the risk of gastric carcinoma mortality was significatively associated with elevated fasting blood glucose, as increased by nearly 32% as added per one standard deviation (Table 2). Simultaneously, the survival analysis showed those patients with elevated fasting blood had significantly shorter survival time (Figure 2). Thus, monitoring and controlling blood glucose could improve the prognosis and prolong the survival time of patients with gastric carcinoma.

In gastric carcinoma, lncRNA was increasingly deemed to an important regulator, which was corrected with larger tumor, larger tumor infiltration, wider metastasis, and shorter survival time [27,28]. The expression level of SNHG8 was consistent with the lncRNA sequencing assay by real-time PCR. Our Previous study had confirmed lncRNA SNHG8 was specifically expressed in an Epstein-Barr virus (EBV) and its expression level was significantly higher in EBV-associated gastric carcinoma. The higher the SNHG8 level was, the later the TNM stage was. SNHG8 acting as a proto-oncogene promoted gastric carcinoma development [17]. Furthermore, lncRNA SNHG8 modulated several functional genes, including TRIM28, NAP1L1 and TRPM7 which affected downstream cancer pathways in gastric cancer. Among them, the overexpression of TRIM28 in gastric cancer was involved in the progression of cancer, and acted as an independent prognostic factor for poor survival [29]. It was interesting that NAP1L1 affected the proliferation of pancreatic neuroendocrine tumor cells via modulation of p57 (Kip2) promoter methylation, whether affected insulin secretion or regulated blood glucose, however, the mechanism had not been elucidated [30]. In our study, we had proved that high SNHG8 expression with shorter survival time (Figure 1) was associated with poor clinical outcome of gastric carcinoma patients (Table 3).

**Table 3:** Multivariate analysis of clinical pathologic characteristic and risk of gastric carcinoma mortality.

	Univariate analysis			Multifactor analysis			
	HR	95%CI	P		HR	95%CI	P*	
Lauren’s Classification	2.84	1.27-6.36	0.011		1.56	0.29-8.51	0.610		
size	3.89	1.93-7.84	<0.001		3.36	1.48-8.96	0.005		
Differentiation	3.13	1.40-7.01	0.005		0.38	0.06-2.54	0.318		
TNM	4.88	1.51-15.74	0.008		2.70	0.60-12.16	0.195		
Distant metastasis	1.35	1.86-8.02	<0.001		2.99	1.19-7.50	0.020		
LNM	6.13	1.90-19.76	0.002		0.92	0.20-4.33	0.917		
SNHG8 expression	1.13	1.06-1.20	<0.001		1.10	1.03-1.19	0.009		
EBV-positive	3.79	2.06-7.00	<0.001		3.40	1.57-7.37	0.002		

Abbreviations: HR=hazard ratio; 95% CI=95% confidence interval; TNM=tumor node metastasis; LNM= lymph node metastasis. The effect estimates were evaluated through Cox regression models. P* : age, gender, and smoking were adjusted.

There was another important result in the interaction of fasting blood glucose and SNHG8 expression. Numerous studies neither used several indicators of epidemiological research interaction, nor did they indicate whether existed multiplication interaction or additive interaction [31]. STROBE suggested that additive interaction analysis should be used to evaluate two risk factors and their joint effect [32]. In this study, we explored the interaction of fasting blood glucose and lncRNA SNGH8 through the COX proportional model [33]. The result demonstrated there was a significant multiplicative interaction between SNHG8 expression and fasting blood glucose (P_multiplicative_=0.005). However, no statistically significant interaction was showed in the additive scale, when relative excess risk due to interaction (RERI) with 95%CI was 0.65(-2.03, 3.33).

There were some limitations in the study. First, all patients were registered from a single center. Second, this study could not be directly extrapolated to whole gastric carcinoma patients, because only cancer patients after radical gastrectomy were recruited. Third, sample size and follow-up time were not sufficient, so it was necessary to design larger sample size and longer follow-up time in the future. Finally, the proportion of EBV positive samples was relatively high, which might leaded to potential bias. For these reasons, large-size and multicenter studies should be designed to evaluate the prognosis predicting value of fasting blood glucose and SNHG8 expression.

In conclusion, our findings convincingly demonstrated that elevated fasting blood glucose and high SNHG8 expression could predict poor prognosis after radical gastrectomy for gastric cancer patients. It suggested that we could introduce lncRNA SNHG8 into the clinical practices as a promising diagnostic biomarker, combined with clinical monitoring and controlling blood glucose. In the future, new therapeutic target could be explored to develop appropriate treatment strategy of gastric carcinoma.

## MATERIALS AND METHODS

### Study population

All participants with gastric carcinoma were consecutively selected from Fujian Cancer Hospital from July 2012 to October 2016. A total of 227 patients were enrolled in this study, 8 patients of them were lost to follow up and 2 patients died of the diseases other than gastric carcinoma. As a result, 217 patients were evaluated in the final analysis. The follow-up time ranged from 1.7 months to 51.6 months and the median time was 20.4 months. The final follow-up assessment was finished in January 2017. The conduct of this study was approved by Fujian Cancer Hospital Ethics Committee and informed consent of all patients before recruitment was obtained.

### Eligibility criteria and tissue collection

The diagnosis of gastric carcinoma was confirmed by pathological examination. Gastric carcinoma participants were eligible for inclusion if they underwent radical gastrectomy for the first time. All participants were Han Chinese patients without relative blood relationship. They also had no history of malignant tumors and without preoperative chemotherapy or radiotherapy. Moreover, they must be followed up for more than 1 month. Fresh tissue was immediately stored in liquid nitrogen and then transferred to a -80°C refrigerator. The whole collection and preservation process were operated according to the principle of no enzyme.

### Real-time PCR assay

100 mg frozen fresh tissue was ground into powder and added to 1 mL Trizol, then mRNA was extracted after standing for 10 min. RNA was reversed to cDNA based on the instruction of reverse transcription reagent kit, and stored at - 80°C until RNA extraction. The reaction system was prepared by fluorescence quantitative PCR according to SYBY Green instructions. PCR conditions were as follows: 90 ° C for 10 min, 40 cycles of 90 ° C for 20 s, 60 ° C for 20 s and 72 ° C for 20 s, each sample was performed in triplicate. β-actin: Upstream primer: 5'-GA GAAGC-3', downstream primer: 5' - CCACGCACACTTCA TG-ATGG-3'. PCR primer sequence: SNHG8: upstream primer: 5' - CCCGAGAACCGT-CAGTTTGA-3', downstream primer: 5'- ACACC CGTTTCC CCA - ACTAC-3'.

Total RNA from 217 paired gastric cancer and adjacent tissues was extracted with Prime Script RT reagent Kit (Takara, Japan). Reverse transcription for lncRNAs was performed using Reverse Transcriptase (Takara, Japan). The cDNA template was amplified by real-time PCR using the SYBR Green Master Mix (Roche, USA). Real-time PCR reactions were performed on the Mx3000P system (Agilent, USA).

The gastric carcinoma specimen T-049 was used as a calibrator. Its expression level was set to 1, other expression levels were quantified. Using beta -actin as an internal reference gene, expression levels of lncRNA were standardized. After adjusting the baseline cycle and calculating the threshold, the relative expression level of lncRNA SNHG8 was reckoned through using the comparative Ct method 2^−ΔΔCt^ [34].

### Follow-up evaluation

After discharge, the assessment of patients was followed up in the outpatient clinic every half a year to one year or by telephone or post mail if the patients were missing at the scheduled time. If gastric cancer death occurred, the exact date was recorded from relatives or medical reports. The meaningful clinical outcome was carcinoma specific mortality. The clinical endpoint event was death of gastric carcinoma. Survival time was defined as the time from initial admission to the date of death or the time of the last follow-up. By the end follow-up time of January 2017, 46 patients had died from gastric carcinoma and only 171 patients had survived.

### Patients characteristics

Venous blood was collected after fasting for at least 8 hours. Triglyceride (TG), total cholesterol (TC), high density lipoprotein (HDL), low density lipoprotein (LDL), fasting blood glucose (FBG), and Epstein -Barr virus (EBV) antibody were measured according to the standard of clinical laboratory. Demographic information was collected through questionnaires, including age, gender, weight, height and smoking history. Age was considered as the age on the date admitted to hospital initially. Body mass index (BMI) was defined as body weight divided by the square of body height (kg/m^2^). Smoking referred to never smoking and smoking formerly or currently. The blood pressure was measured three times by mercury sphygmomanometer, with an interval of one minute, and the average was taken as the measurement result.

### Statistical analysis

Statistical analysis was performed through SPSS 22.0 software. Continuous and categorical variables were expressed as median (interquartile range) and count (percentage), respectively. The data between the groups were compared using the Mann Whitney U test or the chi-square test where appropriate. Multivariate statistical analysis was calculated through Cox regression model. Survival analysis was performed using Kaplan-Meier analysis and compared by log-rank method. The interaction was analyzed by Stata14.0 software. COX regression model was used to evaluate the multiplicative interaction. Through the Bootstrap method [35], relative excess risk ratio (RERI), attributable ratio (AP), interaction index (S) and 95% confidence interval (CI) were calculated to evaluate the additive interaction between the two groups. P < 0.05 was considered statistically significant.

## References

[1] Karimi P, Islami F, Anandasabapathy S, Freedman ND, Kamangar F. Gastric cancer: descriptive epidemiology, risk factors, screening, and prevention. Cancer Epidemiol Biomarkers Prev. 2014; 23:700–13. 10.1158/1055-9965.EPI-13-1057 24618998 PMC4019373

[2] Chen W, Zheng R, Baade PD, Zhang S, Zeng H, Bray F, Jemal A, Yu XQ, He J. Cancer statistics in China, 2015. CA Cancer J Clin. 2016; 66:115–32. 10.3322/caac.21338 26808342

[3] Yau TO, Tang CM, Yu J. Epigenetic dysregulation in Epstein-Barr virus-associated gastric carcinoma: disease and treatments. World J Gastroenterol. 2014; 20:6448–56. 10.3748/wjg.v20.i21.6448 24914366 PMC4047330

[4] Takahashi T, Saikawa Y, Kitagawa Y. Gastric cancer: current status of diagnosis and treatment. Cancers (Basel). 2013; 5:48–63. 10.3390/cancers5010048 24216698 PMC3730304

[5] Chen K, Yang D, Li X, Sun B, Song F, Cao W, Brat DJ, Gao Z, Li H, Liang H, Zhao Y, Zheng H, Li M, et al. Mutational landscape of gastric adenocarcinoma in Chinese: implications for prognosis and therapy. Proc Natl Acad Sci USA. 2015; 112:1107–12. 10.1073/pnas.1422640112 25583476 PMC4313862

[6] Rao Kondapally Seshasai S, Kaptoge S, Thompson A, Di Angelantonio E, Gao P, Sarwar N, Whincup PH, Mukamal KJ, Gillum RF, Holme I, Njølstad I, Fletcher A, Nilsson P, et al, and Emerging Risk Factors Collaboration. Diabetes mellitus, fasting glucose, and risk of cause-specific death. N Engl J Med. 2011; 364:829–41. 10.1056/NEJMoa1008862 21366474 PMC4109980

[7] Onitilo AA, Engel JM, Glurich I, Stankowski RV, Williams GM, Doi SA. Diabetes and cancer I: risk, survival, and implications for screening. Cancer Causes Control. 2012; 23:967–81. 10.1007/s10552-012-9972-3 22552844 PMC4138802

[8] Lindkvist B, Almquist M, Bjørge T, Stocks T, Borena W, Johansen D, Hallmans G, Engeland A, Nagel G, Jonsson H, Selmer R, Diem G, Häggström C, et al. Prospective cohort study of metabolic risk factors and gastric adenocarcinoma risk in the Metabolic Syndrome and Cancer Project (Me-Can). Cancer Causes Control. 2013; 24:107–16. 10.1007/s10552-012-0096-6 23149498

[9] Ge Z, Ben Q, Qian J, Wang Y, Li Y. Diabetes mellitus and risk of gastric cancer: a systematic review and meta-analysis of observational studies. Eur J Gastroenterol Hepatol. 2011; 23:1127–35. 10.1097/MEG.0b013e32834b8d73 21934509

[10] de Jong RG, Peeters PJ, Burden AM, de Bruin ML, Haak HR, Masclee AA, de Vries F, Janssen-Heijnen ML. Gastrointestinal cancer incidence in type 2 diabetes mellitus; results from a large population-based cohort study in the UK. Cancer Epidemiol. 2018; 54:104–11. 10.1016/j.canep.2018.04.008 29705628

[11] Hu D, Peng F, Lin X, Chen G, Zhang H, Liang B, Ji K, Lin J, Chen LF, Zheng X, Niu W. Preoperative Metabolic Syndrome Is Predictive of Significant Gastric Cancer Mortality after Gastrectomy: The Fujian Prospective Investigation of Cancer (FIESTA) Study. EBioMedicine. 2017; 15:73–80. 10.1016/j.ebiom.2016.12.004 27979733 PMC5233804

[12] Kapranov P, Cheng J, Dike S, Nix DA, Duttagupta R, Willingham AT, Stadler PF, Hertel J, Hackermüller J, Hofacker IL, Bell I, Cheung E, Drenkow J, et al. RNA maps reveal new RNA classes and a possible function for pervasive transcription. Science. 2007; 316:1484–88. 10.1126/science.1138341 17510325

[13] Hu JJ, Song W, Zhang SD, Shen XH, Qiu XM, Wu HZ, Gong PH, Lu S, Zhao ZJ, He ML, Fan H. HBx-upregulated lncRNA UCA1 promotes cell growth and tumorigenesis by recruiting EZH2 and repressing p27Kip1/CDK2 signaling. Sci Rep. 2016; 6:23521. 10.1038/srep23521 27009634 PMC4806364

[14] Wang J, Qiu M, Xu Y, Li M, Dong G, Mao Q, Yin R, Xu L. Long noncoding RNA CCAT2 correlates with smoking in esophageal squamous cell carcinoma. Tumour Biol. 2015; 36:5523–28. 10.1007/s13277-015-3220-x 25677908

[15] Liu J, Yang C, Gu Y, Li C, Zhang H, Zhang W, Wang X, Wu N, Zheng C. Knockdown of the lncRNA SNHG8 inhibits cell growth in Epstein-Barr virus-associated gastric carcinoma. Cell Mol Biol Lett. 2018; 23:17. 10.1186/s11658-018-0070-8 29736176 PMC5924468

[16] Huang T, Ji Y, Hu D, Chen B, Zhang H, Li C, Chen G, Luo X, Zheng XW, Lin X. SNHG8 is identified as a key regulator of epstein-barr virus(EBV)-associated gastric cancer by an integrative analysis of lncRNA and mRNA expression. Oncotarget. 2016; 7:80990–1002. 10.18632/oncotarget.13167 27835598 PMC5348371

[17] Chen BZ, Lin XD, Chen G, Hu D, Zhu Q, Shi Y, Wang XJ, Jin SF, Wang HF, Zheng XW. [Expression of long non-coding RNA SNHG8 in Epstein-Barr virus-related gastric cancer and clinical outcome]. Zhonghua Bing Li Xue Za Zhi. 2017; 46:84–87. 28173665 10.3760/cma.j.issn.0529-5807.2017.02.004

[18] Ouyang H, Shiwaku HO, Hagiwara H, Miura K, Abe T, Kato Y, Ohtani H, Shiiba K, Souza RF, Meltzer SJ, Horii A. The insulin-like growth factor II receptor gene is mutated in genetically unstable cancers of the endometrium, stomach, and colorectum. Cancer Res. 1997; 57:1851–54. 9157973

[19] Djiogue S, Nwabo Kamdje AH, Vecchio L, Kipanyula MJ, Farahna M, Aldebasi Y, Seke Etet PF. Insulin resistance and cancer: the role of insulin and IGFs. Endocr Relat Cancer. 2013; 20:R1–17. 10.1530/ERC-12-0324 23207292

[20] Matsubara J, Yamada Y, Hirashima Y, Takahari D, Okita NT, Kato K, Hamaguchi T, Shirao K, Shimada Y, Shimoda T. Impact of insulin-like growth factor type 1 receptor, epidermal growth factor receptor, and HER2 expressions on outcomes of patients with gastric cancer. Clin Cancer Res. 2008; 14:3022–29. 10.1158/1078-0432.CCR-07-1898 18483367

[21] Matsubara J, Hirashima Y, Yamada Y. [Impact of HER2, EGFR, IGF-1R, and VEGFR expressions on the outcome of chemotherapy for advanced gastric cancer]. Gan To Kagaku Ryoho. 2010; 37:1489–96. 20716873

[22] Ge J, Chen Z, Wu S, Chen J, Li X, Li J, Yin J, Chen Z. Expression levels of insulin-like growth factor-1 and multidrug resistance-associated protein-1 indicate poor prognosis in patients with gastric cancer. Digestion. 2009; 80:148–58. 10.1159/000226089 19713703

[23] Nakae J, Kido Y, Accili D. Distinct and overlapping functions of insulin and IGF-I receptors. Endocr Rev. 2001; 22:818–35. 10.1210/edrv.22.6.0452 11739335

[24] Salisbury TB, Tomblin JK. Insulin/Insulin-like growth factors in cancer: new roles for the aryl hydrocarbon receptor, tumor resistance mechanisms, and new blocking strategies. Front Endocrinol (Lausanne). 2015; 6:12. 10.3389/fendo.2015.00012 25699021 PMC4313785

[25] Godsland IF. Insulin resistance and hyperinsulinaemia in the development and progression of cancer. Clin Sci (Lond). 2009; 118:315–32. 10.1042/CS20090399 19922415 PMC2782313

[26] Samani AA, Yakar S, LeRoith D, Brodt P. The role of the IGF system in cancer growth and metastasis: overview and recent insights. Endocr Rev. 2007; 28:20–47. 10.1210/er.2006-0001 16931767

[27] Fang XY, Pan HF, Leng RX, Ye DQ. Long noncoding RNAs: novel insights into gastric cancer. Cancer Lett. 2015; 356:357–66. 10.1016/j.canlet.2014.11.005 25444905

[28] Nasrollahzadeh-Khakiani M, Emadi-Baygi M, Schulz WA, Nikpour P. Long noncoding RNAs in gastric cancer carcinogenesis and metastasis. Brief Funct Genomics. 2017; 16:129–45. 27122631 10.1093/bfgp/elw011

[29] Fitzgerald S, Sheehan KM, O’Grady A, Kenny D, O’Kennedy R, Kay EW, Kijanka GS. Relationship between epithelial and stromal TRIM28 expression predicts survival in colorectal cancer patients. J Gastroenterol Hepatol. 2013; 28:967–74. 10.1111/jgh.12157 23425061

[30] Schimmack S, Taylor A, Lawrence B, Alaimo D, Schmitz-Winnenthal H, Büchler MW, Modlin IM, Kidd M. A mechanistic role for the chromatin modulator, NAP1L1, in pancreatic neuroendocrine neoplasm proliferation and metastases. Epigenetics Chromatin. 2014; 7:15. 10.1186/1756-8935-7-15 25071868 PMC4112619

[31] Knol MJ, VanderWeele TJ. Recommendations for presenting analyses of effect modification and interaction. Int J Epidemiol. 2012; 41:514–20. 10.1093/ije/dyr218 22253321 PMC3324457

[32] Vandenbroucke JP, von Elm E, Altman DG, Gøtzsche PC, Mulrow CD, Pocock SJ, Poole C, Schlesselman JJ, Egger M, and STROBE Initiative. Strengthening the Reporting of Observational Studies in Epidemiology (STROBE): explanation and elaboration. Epidemiology. 2007; 18:805–35. 10.1097/EDE.0b013e3181577511 18049195

[33] Qiu H, Yu IT, Wang XR, Fu ZM, Tse SL. [Study on the interaction under logistic regression modeling]. Zhonghua Liu Xing Bing Xue Za Zhi. 2008; 29:934–37. 19173863

[34] Lin XD, Chen SQ, Qi YL, Zhu JW, Tang Y, Lin JY. Overexpression of thrombospondin-1 in stromal myofibroblasts is associated with tumor growth and nodal metastasis in gastric carcinoma. J Surg Oncol. 2012; 106:94–100. 10.1002/jso.23037 22231149

[35] Andersson T, Alfredsson L, Källberg H, Zdravkovic S, Ahlbom A. Calculating measures of biological interaction. Eur J Epidemiol. 2005; 20:575–79. 10.1007/s10654-005-7835-x 16119429

