# Correction: The fasting blood glucose and long non-coding RNA SNHG8 predict poor prognosis in patients with gastric carcinoma after radical gastrectomy

**DOI:** 10.18632/aging.101755

**Published:** 2018-12-29

**Authors:** Yunchai Lin, Dan Hu, Qiang Zhou, Xiandong Lin, Jinxiu Lin, Feng Peng

**Affiliations:** 1 Department of Cardiology, The First Affiliated Hospital of Fujian Medical University, Fuzhou, Fujian, China; 2 Department of Pathology, Fujian Provincial Cancer Hospital, The Affiliated Hospital of Fujian Medical University, Fuzhou, Fujian, China; 3 Department of Endocrinology, The Second Hospital of Fuzhou, The Affiliated Hospital of Xiamen University, Fuzhou, Fujian, China; * Equal contribution

Original article: Aging (Albany NY) 2018; 10: 2646-2656

**This article has been corrected:** The authors requested to change the second affiliation for Drs. Dan Hu and Xiandong Lin. The correct affiliation is provided below. The authors declare that this correction does not change the results or conclusions of this paper. The authors sincerely apologize for this error.

**^2^Department of Pathology, Fujian Cancer Hospital and Fujian Medical University Cancer Hospital, Fuzhou, Fujian, China**

